# Perceived risk and distress related to COVID-19 in healthcare versus non-healthcare workers of Pakistan: a cross-sectional study

**DOI:** 10.1186/s12960-022-00705-4

**Published:** 2022-01-22

**Authors:** Adeel Abid, Hania Shahzad, Hyder Ali Khan, Suneel Piryani, Areeba Raza Khan, Fauziah Rabbani

**Affiliations:** 1 Medical College, Aga Khan University, Karachi, Pakistan; 2 Department of Community Health Sciences, Aga Khan University, Karachi, Pakistan; 3 Office of Research & Graduate Studies, Aga Khan University, Karachi, Pakistan

**Keywords:** COVID-19, Healthcare workers, Risk perception, Anxiety, Depression, Psychological distress

## Abstract

**Background:**

Healthcare workers (HCWs) have found themselves and their families more susceptible to contracting COVID-19. This puts them at a higher risk of psychological distress, which may compromise patient care. In this study, we aim to explore the risk perceptions and psychological distress between HCWs and non-healthcare workers (NHCWs) in Pakistan.

**Methods:**

A cross-sectional study was conducted using an online self-administered questionnaire. Psychological distress was assessed through The Hospital Anxiety and Depression Scale (HADS). Comparisons were made between HCWs (front/backend, students/graduates) and NHCWs related to risk perceptions and stress levels related to COVID-19. Following tests for normality (Shapiro–Wilk test), variables that fulfilled the normality assumption were compared using the independent samples *t*-test, while for other variables Mann–Whitney *U*-test was employed. Pearson Chi-square test was used to compare categorical data. Multiple logistic regression techniques examined the association of participant age, gender, household income, and the presence of COVID-19 symptoms with depression and anxiety levels.

**Results:**

Data from 1406 respondents (507 HCWs and 899 NHCWs) were analyzed. No significant difference was observed between HCWs and NHCWs’ perception of susceptibility and severity towards COVID-19. While healthcare graduates perceived themselves (80% graduates vs 66% students, *p*-value 0.011) and their family (82% graduates vs 67% students, *p*-value 0.008) to be more susceptible to COVID-19, they were less likely to experience depression than students. Frontline HCWs involved in direct patient care perceived themselves (83% frontline vs. 70% backend, *p*-value 0.003) and their family (84% frontline vs. 72% backend, *p*-value 0.006) as more susceptible to COVID-19 than backend healthcare professionals. Over half of the respondents were anxious (54% HCWs and 55% NHCWs). Female gender, younger age, lower income, and having COVID-19 related symptoms had a significant effect on the anxiety levels of both HCWs and NHCWs.

**Conclusion:**

Frontline HCWs, young people, women, and individuals with lower income were at a higher risk of psychological distress due to the pandemic. Government policies should thus be directed at ensuring the mental well-being of frontline HCWs and improving their satisfaction to strengthen the health care delivery system. The findings suggest the need to provide mental health support for health workers.

**Supplementary Information:**

The online version contains supplementary material available at 10.1186/s12960-022-00705-4.

## Background

The world has grappled with COVID-19 since the first case was diagnosed in Wuhan, China [1]. This has resulted in a global socio-economic crisis and challenged healthcare systems throughout the world. Healthcare workers (HCWs) are at constant risk of many infectious diseases due to the nature of their occupation as evidenced by the previous severe acute respiratory syndrome (SARS) and SARS-coronavirus (SARS-CoV) outbreaks [2–4]. A major concern in the country is the impact of COVID-19 on healthcare workers (HCWs), who are at high risk during novel disease outbreaks. HCWs around the world are at the forefront in screening, quarantining, and managing actual and suspected COVID-19 patients, creating awareness about risks, and advocating for preventive measures [5]. However, not all HCWs are at an equal risk of contracting the COVID-19 infection. In a recent systematic review, frontline HCWs with increased face-to-face interactions, exposure to COVID-19 positive patients, and those working in health facilities designated as treatment centers were shown to have higher infection rates compared to non-front line HCWs [6].

Increased anxiety and depression among frontline healthcare professionals is also a common feature in epidemics [7, 8]. Experience from severe acute respiratory syndrome (SARS) and H1N1 outbreaks highlights how large-scale epidemics and pandemics have a severe psychological strain on healthcare professionals, especially those working on the frontline [9, 10]. Compared to the general public, HCWs face more personal worries such as greater infection risk to self and others and psychological concerns regarding the well-being of their family members [11]. There are disparities in terms of the psychological impact of COVID-19 on HCWs depending on their level of patient care. A study among healthcare professionals in a tertiary infectious disease hospital for COVID-19 in China also revealed a high incidence of anxiety and stress disorders among frontline medical staff, with a higher incidence of anxiety among nurses than doctors [12].

The utility of online surveys to assess psychological distress caused by COVID-19 has proven to be efficient and effective in recruiting large and diverse samples of literate respondents. Social media platforms have thus aided in scientific data collection when other methods of recruitment are no longer safe, practical, and economically feasible. One such study in Hong Kong was developed through Google Forms, which was emailed to councilors of the 18 districts in the region. Although the authors acknowledge the under-sampling of individuals without internet access, Google Forms was the only feasible tool for data collection during this initial phase of COVID to understand early community response to the pandemic [13].

In contrast to Hong Kong, the scale of the COVID-19 health crisis is a bigger concern in a resource-limited country like Pakistan, where psychological morbidity especially in the healthcare community may compromise the quality of care and health care services. Prevention remains the mainstay in the treatment and containment of the pandemic, requiring people at large to practice COVID-19 mitigating behaviors. As a result, it also becomes important to study the public response in the early phase of the pandemic when the nature of the threat is usually ambiguous. This can help to develop strategies to cope with the pandemic. Identifying vulnerable subgroups for psychological distress will help in strengthening health service delivery with targeted interventions.

Unlike Hong Kong which had experience with past pandemics like SARS, it was the first time Pakistan was exposed to a pandemic which began in the first quarter of 2020. Hence, we used the same tool as was used in the study conducted in Hong Kong [13] to understand the first impressions, behavioral responses, stress levels of various population subsets (including both health and non-health workforce) to devise appropriate policy interventions.

Using an online approach, this study aims to assess perceived severity, susceptibility, and anxiety levels of HCWS in comparison to non-healthcare workers (NHCWs). Furthermore, we explore vulnerable subgroups in the healthcare population with regard to training status, age, gender, income, and level of patient care. This study is unique in Pakistan for targeting HCWs and NHCWs during the early stage of the pandemic.

## Materials and methods

### Study design, setting, and data collection instrument

A cross-sectional online survey was carried out in May 2020 using a survey tool developed over Google Forms (please refer to Additional file 1). This is a reliable tool as it has been used in the previously mentioned study, conducted in Hong Kong [13]. For contextual relevance, the tool was pretested with 10 individuals who had internet access. Of these, five were HCWs who either had or were working towards a health-related degree and five were NHCWs—literate individuals but not involved in the medical or allied fields. Following the pretesting, a link to the final questionnaire was shared through social media channels of the Aga Khan University (AKU), Pakistan, which included Facebook, Twitter, and LinkedIn. The survey link was also reposted on the Facebook page of AKU. The online survey link remained active for 2 weeks.

Data were collected through an online self-administered semi-structured questionnaire designed separately in English and Urdu (national language). Respondents were directed to questions regarding their demographics (including gender, age, level of education, household income, permanent city of residence) and recent travel history. This was followed by questions about their health status in the past 14 days and whether they experienced any symptoms of illness. Next, they were asked to rate the severity of COVID-19 symptoms and their perceived chance of survival if infected with the disease. This was then followed by questions on how likely one considered their families and themselves to be infected with COVID-19 if no preventive measures were taken. Responses were captured using a five-point Likert Scale to provide a range of responses to a given question or statement [14]. Five categories of responses were used, ranging from strongly disagree to strongly agree.

Participants’ mental health was assessed using the validated Hospital Anxiety and Depression Scale (HADS). This scale has two subscales to assess for anxiety (HADS-A) and depression (HADS-D). Each subscale has a minimum possible score of 0 and a maximum of 21. A score of eight or above indicates anxiety or depression [14]. Respondents were also asked about the psychological impact of COVID-19 on their job, personal life, sleep, and eating habits.

HCWs also provided information on their field of work, educational status (graduate or student), level of patient care (frontline vs. backend), and their perception of governmental measures to combat COVID-19.

### Study participants

Eligibility for enrollment in the study was assessed on the first page of the Google Form. Respondents were recruited as study participants if they were aged 18 or above, were residing in Pakistan for at least 5 days a week over the last month (April to May 2020), and were willing to participate in the survey. Participants who met the above-stated eligibility criteria, and consented to participate were able to further navigate the study tool. Respondents found ineligible or those not willing to consent were redirected to a thank you message, and further access to the tool was halted.

After going through the screening questions and providing consent, participants were categorized into NHCWs and HCWs. Those without basic (Bachelor level) training in any health or allied field were categorized as NHCWs, whereas respondents having formal training (students or graduates) in Medicine, Nursing, Pharmacy, Dentistry, Physiotherapy, Laboratory Technology or Allied Health Sciences including but not limited to homeopathy and *Hikmat* (alternative systems of medicine) were categorized as HCWs. While this definition excluded support staff working in health, such as ward attendants, laundry staff, as well as food and catering services; evidence suggests that the demographic characteristics of the latter group may preclude internet accessibility, or may indicate a level of language comprehension below what is required to respond to such a study [15, 16]. Perceptions of junior ancillary staff in hospitals are better assessed through direct face-to-face interactions/interviews, especially when participants have variable ability to read and understand questions [17]. Conducting such face-to-face interviews was outside the realm of this study due to the social distancing requirements imposed by the pandemic.

HCWs were further categorized into frontline and backend HCWs. Frontline HCWs included all those professionals who are involved in patients’ direct bedside medical care. Backend HCWs included those who are currently not involved in clinical bedside care, including undergraduate students of Medicine, Nursing, and HCWs employed in the fields of Pharmacy, Dentistry, Physiotherapy, Laboratory Technology, Allied Health Sciences, etc.

### Statistical analysis

Data collected from respondents were directly stored in Google Spreadsheets and later imported to Microsoft Excel and Statistical Package for the Social Sciences (SPSS) Version 21 (IBM Corp). Data were cleaned, coded, and analyzed using SPSS. Descriptive analyses were performed and results were tabulated as numbers (percentages) for qualitative variables and mean (± standard deviation) for quantitative variables.

Comparisons were made between HCWs (front/backend, students/graduates) and NHCWs related to risk perceptions and stress levels related to COVID-19. Following tests for normality (Shapiro–Wilk test), variables that fulfilled the normality assumption (mean HADS scores) were compared using the independent samples *t*-test, while for other variables (perceived disease susceptibility and severity, the impact of COVID-19 on sleeping/eating/smoking/drug usage habits, satisfaction with government measures) Mann–Whitney *U*-test was employed. Pearson Chi-square test was used to compare categorical data, such as normal versus abnormal HADS scores and adoption of precautionary measures.

Multiple logistic regression techniques were used to further examine the association of anxiety and depression among HCWs vs NHCWs with regard to participant age, gender, household income, and presence of symptoms. All predictors were entered using a stepwise approach to adjust for the effect of confounding. The results of the multivariable analysis are reported as adjusted OR with 95% CI. A two-sided level of significance was used and any association with a *p*-value less than 0.05 was considered statistically significant.

## Results

Among the 507 HCWs and 899 NHCWs, a majority of the respondents were males (53% HCWs and 72% NHCWs), below the age of 35 years (78% HCWs and 61% NHCWs), were permanent residents of Karachi (49% HCWs and 50% NHCWs), and had a household income of less than or equal to Pakistani Rupee (PKR) 40,000 (22% HCWs and 27% NHCWs) (refer to Table 1). More than half of the HCWs (54%) belonged to the field of Medicine. Among healthcare graduates, 36% were currently working in a hospital, ward, or clinic (refer to Table 2). For raw and de-identified data, please refer to Additional file 2.

**Table 1:** Socio-demographic characteristics of the respondents

Characteristics	HCWs *n* = 507 No. (%)	NHCWs *n* = 899 No. (%)
Gender		
Male	269 (53.1)	644 (71.7)
Female	235 (46.3)	243 (27.0)
Prefer not to disclose	3 (0.6)	12 (1.3)
Age		
18–24 years	220 (43.4)	239 (26.6)
25–34 years	173 (34.1)	309 (34.4)
35–44 years	69 (13.6)	213 (23.7)
45–54 years	31 (6.1)	73 (8.1)
55 or above	14 (2.8)	61 (6.8)
Prefer not to disclose	0	4 (0.4)
Education		
Up to Matric/O-Levels		18 (2.0)
Intermediate/A-Levels/International Baccalaureate		110 (12.2)
Post-intermediate: Diploma/Certificate		22 (2.5)
Bachelor or above		743 (82.6)
Prefer not to disclose		6 (0.7)
Household incomes		
PKR 40,000 or below	73 (22.4)	246 (27.4)
PKR 40,001–PKR 80,000	70 (21.4)	176 (19.6)
PKR 80,001–PKR 120,000	50 (15.4)	156 (17.3)
≥ PKR 120,001	56 (17.2)	142 (15.8)
Prefer not to disclose	77 (23.6)	179 (19.9)
Permanent residence		
a. Karachi	249 (49.1)	451 (50.2)
b. Lahore	46 (9.0)	82 (9.0)
c. Islamabad	24 (4.8)	61 (6.8)
d. Peshawar	30 (5.9)	23 (2.6)
e. Quetta	8 (1.6)	7 (0.8)
f. Hyderabad	21 (4.2)	32 (3.6)
g. Other cities of Pakistan	129 (25.4)	243 (27.0)

**Table 2:** HCWs’ field, training status, and current work status (*n* = 507)

Characteristics	No. (%)
Healthcare field	
Medicine	274 (54.0)
Nursing	52 (10.3)
Pharmacy	42 (8.3)
Dentistry	21 (4.1)
Physiotherapy	24 (4.7)
Laboratory Technology or Allied Health Sciences	87 (17.2)
Others	7 (1.4)
Training status	
Student	181 (35.7)
Graduate	326 (64.3)
Current work status of graduate HCWs	
Working in hospital/ward/clinic	117 (35.9)
Working from home (online/telephone, etc.)	49 (15.0)
Working in office setting	28 (8.6)
Unpaid leave	28 (8.6)
Paid leave	18 (5.5)
Not working	86 (26.4)

### Perceived severity and susceptibility for COVID-19

No significant difference was observed between HCWs and NHCWs’ perception of susceptibility and severity towards COVID-19 (Table 3). About three-fourths of the respondents perceived that they (75% HCWs and 71% NHCWs, *p*-value 0.506) and their families (77% HCWs and 71% NHCWs, *p*-value 0.539) might get sick if they do not take preventive measures. Similarly, several respondents considered the symptoms of COVID-19 (if infected) as serious (46% HCWs and 38% NHCWs, *p*-value 0.916). Furthermore, most respondents thought that one could survive a COVID-19 infection (HCWs 70% and NHCWs 66%, *p*-value 0.807).

**Table 3:** Perceived severity and susceptibility for COVID-19

Variable	Perception	HCWs	NHCWs	HCWs vs NHCWs	Healthcare students	Healthcare graduates	Students vs graduates	Frontline HCWs	Backend HCWs	Frontline vs backend
		*n* = 507	*n* = 899		*n* = 181	*n* = 326		*n* = 216	*n* = 290	
		No. (%)	No. (%)	*p*-value*	No. (%)	No. (%)	*p*-value*	No. (%)	No. (%)	*p*-value*
Susceptibility										
1. I might contract the disease if no preventive measure is taken	Agree	382 (75.3)	636 (70.7)	0.506	120 (66.3)	262 (80.4)	**0.011**	179 (82.9)	203 (69.8)	**0.003**
	Neutral	41 (8.1)	128 (14.3)		18 (9.9)	23 (7.1)		17 (7.9)	24 (8.2)	
	Disagree	81 (16.0)	129 (14.3)		42 (23.2)	39 (11.9)		20 (9.2)	61 (21.0)	
	Don’t know	3 (0.6)	6 (0.7)		1 (0.6)	2 (0.6)		0	3 (1.0)	
2. My family might contract the disease if no preventive measure is taken	Agree	389 (76.7)	642 (71.4)	0.539	121 (66.8)	268 (82.2)	**0.008**	181 (83.8)	208 (71.5)	**0.006**
	Neutral	31 (6.1)	116 (12.9)		15 (18.3)	16 (4.9)		10 (4.6)	21 (7.2)	
	Disagree	84 (16.6)	133 (14.8)		44 (24.3)	40 (12.3)		25 (11.6)	59 (20.3)	
	Don’t know	3 (0.6)	8 (0.9)		1 (0.6)	2 (0.6)		0	3 (1.0)	
3. I might contract COVID-19 if one of my family members tests positive for the disease	Agree	350 (69.3)	594 (66.1)	0.559	118 (65.6)	232 (71.4)	0.637	158 (73.5)	192 (66.3)	0.138
	Neutral	60 (11.9)	140 (15.5)		18 (10.0)	42 (12.9)		24 (11.2)	36 (12.4)	
	Disagree	79 (15.6)	139 (15.5)		38 (21.1)	41 (12.6)		27 (12.5)	52 (17.9)	
	Don’t know	16 (3.2)	26 (2.9)		6 (3.3)	10 (3.1)		6 (2.8)	10 (3.4)	
Severity										
1. Seriousness of symptoms caused by SARS-CoV 19	Severe	232 (45.8)	342 (38.0)	0.916	96 (53.0)	136 (41.7)	**0.04**	93 (43.1)	139 (47.9)	**0.045**
	Neutral	148 (29.2)	192 (21.4)		45 (24.9)	103 (31.6)		64 (29.6)	84 (28.9)	
	Not Severe	82 (16.1)	127 (14.1)		23 (12.7)	59 (18.1)		44 (20.4)	38 (13.0)	
	Don’t know	45 (8.9)	238 (26.5)		17 (9.4)	28 (8.6)		15 (6.9)	30 (10.2)	
2. Chance of survival if infected with COVID-19	High	356 (70.2)	596 (66.3)	0.807	124 (68.5)	232 (71.2)	0.46	147 (68.1)	209 (71.8)	0.188
	Neutral	105 (20.7)	173 (19.2)		40 (20.1)	65 (19.9)		49 (22.7)	56 (19.3)	
	Not high	34 (6.7)	51 (5.7)		13 (7.2)	21 (6.4)		17 (7.9)	17 (5.8)	
	Don’t know	12 (2.4)	79 (8.8)		4 (2.2)	8 (2.5)		3 (1.3)	9 (3.1)	

Bold figures are significant

Categories were merged, so “agree/severe/high” and “strongly agree/very severe/very high” were merged into category “agree/severe/high”, and categories “disagree/not severe/not high” and “strongly disagree/not severe at all/not high at all” were merged into “disagree/not severe/not high”

^*^Mann–Whitney test

A significant difference was seen between the healthcare students’ and graduates’ perception of susceptibility and severity towards COVID-19. Healthcare graduates perceived themselves (80% graduates vs 66% students, *p*-value 0.011) and their families (82% graduates vs 67% students, *p*-value 0.008) to be more susceptible to COVID-19 than the healthcare students. Similarly, compared to students, fewer graduates perceived the disease to be severe (53% students vs. 42% graduates, *p*-value 0.040).

A significant difference was also seen between frontline and backend HCWs’ perception of their susceptibility towards COVID-19. Frontline HCWs perceived themselves (83% frontline vs. 70% backend, *p*-value 0.003) and their family (84% frontline vs. 72% backend, *p*-value 0.006) as being more susceptible to COVID-19 than backend HCWs. However, compared to those on the frontline, more backend HCWs perceived the disease to be severe (*p*-value 0.045) (refer to Table 3).

### Psychological distress in HCWs and NHCWs

More than half of the respondents were found to be either anxious, (54% HCWs and 55% NHCWs, *p*-value 0.697) or depressed (54% HCWs and 57% NHCWs, *p*-value 0.282) as indicated by the HADS scores. No significant difference was seen in the anxiety and depression levels of HCWs and NHCWs (Table 4).

**Table 4:** Perceived psychological impact of COVID-19

Variables	HCWs	NHCWs	HCWs vs NHCWs	HCW students	HCW graduates	Students vs graduates	Frontline HCWs	Backend HCWs	Frontline vs backend
	*n* = 507	*n* = 899		*n* = 181	*n* = 326		*n* = 216	*n* = 290	
	No. (%)	No. (%)	*p*-value	No. (%)	No. (%)	*p*-value	No. (%)	No. (%)	*p*-value
Anxiety (HADS-A Score cut-off ≥ 6)									
Normal	235 (46.4)	407 (45.3)	0.697*	78 (43.1)	157 (48.2)	0.273*	96 (44.4)	139 (47.8)	0.458*
Abnormal	272 (53.6)	492 (54.7)		103 (56.9)	169 (51.8)		120 (55.6)	152 (52.2)	
Mean (SD)	6.07 (3.56)	6.34 (3.65)	0.177^†^	6.25 (3.33)	5.98 (3.69)	0.409^†^	6.9 (3.60)	5.98 (3.54)	0.509^†^
Depression (HADS-D Score cut-off ≥ 8)									
Normal	235 (46.4)	390 (43.4)	0.282*	68 (37.6)	167 (51.2)	0.003*	109 (50.5)	126 (43.3)	0.110*
Abnormal	272 (53.6)	509 (56.6)		113 (62.4)	159 (48.8)		107 (49.5)	165 (56.7)	
Mean (SD)	7.97 (3.69)	8.26 (3.83)	0.163^†^	8.40 (3.45)	7.72 (3.80)	**0.047**^†^	7.69 (3.92)	8.18 (3.50)	0.139^†^
COVID-19 will affect my job									
Agree	315 (62.3)	529 (58.8)	0.592^‡^	105 (58.3)	210 (64.4)	0.844^‡^	146 (67.6)	169 (58.4)	0.250^‡^
Neutral	65 (12.8)	167 (18.6)		28 (15.6)	37 (11.4)		26 (12.0)	39 (13.4)	
Disagree	113 (22.3)	168 (18.7)		39 (21.7)	74 (22.7)		41 (19.0)	72 (24.8)	
Don’t know	13 (2.6)	35 (3.9)		8 (4.4)	5 (1.5)		3 (1.4)	10 (3.4)	
COVID-19 will affect my personal life									
Agree	331 (65.3)	561 (62.4)	0.877^‡^	112 (61.9)	219 (67.2)	0.260^‡^	161 (74.5)	170 (58.4)	**< 0.001**^‡^
Neutral	70 (13.8)	176 (19.6)		26 (14.4)	44 (13.5)		23 (10.6)	47 (16.2)	
Disagree	101 (19.9)	149 (16.6)		41 (22.6)	60 (18.4)		31 (14.4)	70 (24.1)	
Don’t know	5 (1.0)	13 (1.4)		2 (1.1)	3 (0.9)		1 (0.5)	4 (1.3)	
COVID-19 has affected my sleeping pattern									
Agree	175 (34.5)	374 (41.6)	0.060^‡^	63 (34.8)	112 (34.4)	0.324^‡^	79 (36.6)	96 (33.0)	0.616^‡^
Neutral	90 (17.8)	150 (16.7)		33 (18.2)	57 (17.5)		33 (15.3)	57 (19.6)	
Disagree	220 (43.4)	351 (39.0)		74 (40.9)	146 (44.8)		97 (44.9)	123 (42.3)	
Don’t know	22 (4.3)	24 (2.7)		11 (6.1)	11 (3.3)		7 (3.2)	15 (5.1)	
COVID-19 has affected my eating habits									
Agree	167 (33.0)	335 (37.3)	0.108^‡^	68 (37.6)	99 (30.5)	0.100^‡^	69 (32.2)	98 (33.7)	0.302^‡^
Neutral	88 (17.4)	165 (18.4)		29 (16.0)	59 (18.2)		36 (16.7)	52 (17.9)	
Disagree	236 (46.6)	378 (42.0)		77 (42.5)	159 (48.8)		105 (48.8)	131 (45.0)	
Don’t know	15 (3.0)	21 (2.3)		7 (3.9)	8 (2.5)		5 (2.3)	10 (3.4)	
I might start/increase smoking cigarettes									
Agree	54 (10.7)	78 (8.7)	0.461^‡^	20 (11.1)	34 (10.4)	0.675	27 (12.5)	27 (9.3)	0.429^‡^
Neutral	32 (6.3)	89 (9.9)		10 (5.5)	19 (5.8)		13 (6.0)	19 (6.5)	
Disagree	397 (78.3)	692 (77.0)		141 (77.9)	256 (78.5)		166 (76.9)	231 (79.4)	
Don’t know	24 (4.7)	40 (4.4)		10 (5.5)	14 (4.3)		10 (4.6)	14 (4.8)	
I might start/increase the use of recreational drugs									
Agree	34 (6.7)	38 (4.2)	0.154^‡^	11 (6.1)	23 (7.1)	0.393	19 (8.8)	15 (5.2)	0.307‡
Neutral	31 (6.1)	61 (6.8)		6 (3.3)	25 (7.6)		12 (5.6)	19 (6.5)	
Disagree	419 (82.6)	757 (84.2)		153 (84.5)	266 (81.6)		177 (81.9)	242 (83.1)	
Don’t know	23 (4.6)	43 (4.8)		11 (6.1)	12 (3.7)		8 (3.7)	15 (5.2)	

Bold figures are significant

Percentages of categories “agree/ strongly agree” were merged into category “agree”, and categories “disagree/strongly disagree” were merged into “disagree”

^*^Pearson Chi-square test

^†^Independent-samples *t*-test

^‡^Mann–Whitney test

The incidence of depression was significantly higher among healthcare students compared to healthcare graduates (HADS-D: Mean (SD): 8.40 (3.45) in students; 7.72 (3.80) in graduates, *p*-value 0.047). Around 62% of healthcare students and 49% of graduates had depression (*p*-value 0.003).

A significant difference was noted between frontline and backend HCWs’ perceptions about the impact of COVID-19 on their personal life (75% frontline vs. 58% backend HCWs, *p*-value < 0.001). However, no significant difference was reported between HCWs’ and NHCWs’ perceived impact of COVID-19 on their jobs, personal life, sleeping pattern, and or eating habits (refer to Table 4).

### Predictors of psychological distress in HCWs and NHCWs

Gender, age, and presence of symptoms had significant associations with anxiety among HCWs (Table 5). The odds of female HCWs experiencing anxiety were nearly twice as compared to male HCWs (aOR: 2.34, 95% CI 1.37–3.99, *p*-value 0.002). The odds of younger HCWs (age between 25 and 34 years) experiencing anxiety were nearly three times that of HCWs above the age of 55 (aOR 3.44, 95% CI 1.30–9.09, *p*-value: 0.013). The odds of HCWs with COVID-19 related symptoms experiencing anxiety was 2.09 times compared to HCWs without symptoms (aOR: 2.09, 95% CI 1.01–4.32, *p*-value: 0.046) (refer to Table 5).

**Table 5:** Predictors of anxiety and depression

Variable	Anxiety				Depression			
	HCWs (*n* = 507)		NHCWs (*n* = 899)		HCWs		NHCWs	
	aOR^a^ (95% CI)	*p*-value	aOR^a^ (95% CI)	*p*-value	aOR^a^ (95% CI)	*p*-value	aOR^a^ (95% CI)	*p*-value
Gender								
Female	2.34 (1.37–3.99)	**0.002**	1.62 (1.12–2.35)	**0.010**	1.53 (0.90–2.58)	0.115	1.41 (0.98–2.02)	0.065
Male	Reference		Reference		Reference		Reference	
Age								
18–24 years	3.52 (1.19–10.42)	**0.023**	2.53 (1.51–4.23)	**< 0.001**	1.00 (0.37–2.71)	1.000	1.38 (0.84–2.27)	0.209
25–34 years	3.44 (1.30–9.09)	**0.013**	2.84 (1.75–4.62)	**< 0.001**	1.72 (0.72–4.11)	0.219	1.26 (0.79–2.01)	0.325
35–44 years	4.68 (1.63–13.47)	**0.004**	2.21 (1.31–3.70)	**0.003**	1.01 (0.39–2.64)	0.978	1.66 (1.00–2.76)	**0.050**
45 years or above	Reference		Reference		Reference		Reference	
Household incomes								
≤ PKR 60,000	1.30 (0.62–2.71)	0.491	1.61 (1.06–2.43)	**0.024**	1.12 (0.54–2.32)	0.762	1.58 (1.06–2.36)	**0.026**
PKR 60,001–PKR 120,000	1.35 (0.64–2.84)	0.430	2.22 (1.42–3.48)	**< 0.001**	1.25 (0.61–2.57)	0.548	2.29 (1.48–3.54)	**< 0.001**
> PKR 120,000	Reference		Reference		Reference		Reference	
Presence of COVID-19 related symptoms								
Yes	2.09 (1.01–4.32)	**0.046**	1.98 (1.34–2.94)	**0.001**	2.72 (1.34–5.55)	**0.006**	1.41 (0.96–2.06)	0.077
No	Reference		Reference		Reference		Reference	

Bold figures are significant

^a^Adjusted for gender, age, household income, and presence of COVID-19 related symptoms

Similarly, gender, age, household income, and presence of symptoms were positively associated with anxiety among NHCWs. The odds of female NHCWs experiencing anxiety was 1.62 times more than their male counterparts (aOR: 1.62, 95% CI 1.12–2.35, *p*-value 0.010). The odds of younger NHCWs (25–34 years) experiencing anxiety were nearly three times more than NHCWs of 45 years or above (aOR 2.84, 95% CI 1.75–4.62, *p*-value: < 0.001). The odds of NHCWs with an income level of 60,001–120,000 PKR to experience anxiety was 2.22 times more than NHCWs having household income ≥ PKR 120,000 PKR (aOR: 2.22, 95% CI 1.42–3.48, *p*-value: < 0.001). NHCWs having COVID-19 related symptoms were 1.98 times more likely to have anxiety than HCWs without symptoms (aOR: 1.98; 95% CI 1.34–2.94, *p*-value: 0.001) (refer to Table 5).

Furthermore, the presence of symptoms was positively associated with depression among HCWs (aOR: 2.72; 95% CI 1.34–5.55, *p*-value: 0.006). Household income had a positive association with depression among NHCWs. The odds of NHCWs with an income level of 60,001–120,000 PKR experiencing depression was nearly twice in comparison to NHCWs having household income > PKR 120,000 PKR (aOR: 2.29, 95% CI 1.48–3.54, *p*-value: < 0.001) (refer to Table 5).

### Adoption of precautionary measures

Significantly more HCWs reported wearing face masks (94% HCWs vs. 91% NHCWs, *p*-value 0.012), avoiding visiting meat shops or markets (77% HCWs vs. 66% NHCWs, *p*-value < 0.001) than NHCWs. Moreover, significantly fewer HCWs reported that they refrain from going to hospitals or clinics (60% HCWs vs. 81% NHCWs, *p*-value < 0.001) and work (55% HCWs vs. 66% NHCWs, *p*-value < 0.001) compared to NHCWs.

Additionally, there was a significant difference between healthcare students’ and graduates’ adoption of some precautionary measures such as washing their hands with soap/sanitizer frequently (96% students vs. 99% graduates, *p*-value 0.001), avoiding going out (87% students vs. 73% graduates, *p*-value 0.003), and refraining from going to hospital or clinic (80% students vs. 50% graduates, *p*-value < 0.001).

Similarly, a significant difference was noted between frontline and backend HCWs in the adoption of some precautionary measures such as refraining from going to hospital or clinic (45% frontline vs. 72% backend, *p*-value < 0.001) and avoiding going to work (37% frontline vs. 68% backend, *p*-value < 0.00). Frontline workers were more likely to report washing their hands with soap/sanitizer frequently (100% frontline vs. 97% backend, *p*-value 0.009) (refer to Table 6).

**Table 6:** Adoption of precautions by the respondents (number of respondents answering “yes”)

Precautions	HCWs	NHCWs	HCWs vs NHCWs	healthcare students	Healthcare graduates	Students vs graduates	Frontline NHCWs	Backend NHCWs	Frontline vs backend
	*n* = 507	*n* = 899		*n* = 181	*n* = 326		*n* = 216	*n* = 290	
	No. (%)	No. (%)	*p*-value*	No. (%)	No. (%)	*p*-value*	No. (%)	No. (%)	*p*-value*
1. Wear face masks	475 (93.7)	815 (90.7)	**0.012**	161 (89.0)	314 (96.3)	0.082	208 (96.3)	267 (91.8)	0.344
2. Wash hands frequently (with soap or hand sanitizer)	497 (98.0)	884 (98.3)	0.756	173 (95.6)	324 (99.4)	**0.001**	215 (99.5)	282 (96.9)	**0.009**
3. Avoid contacting people who have fever or respiratory symptoms	470 (92.7)	825 (91.8)	0.597	163 (90.1)	307 (94.2)	**0.047**	203 (94.0)	267 (91.8)	0.212
4. Avoid going out	397 (78.3)	679 (75.5)	0.089	158 (87.3)	239 (73.3)	**0.003**	164 (75.9)	233 (80.1)	0.685
5. Avoid going to meat shops/market	391 (77.1)	592 (65.9)	**< 0.001**	142 (78.5)	249 (76.4)	0.291	172 (79.6)	219 (75.3)	0.330
6. Avoid going to hospital or clinic	306 (60.4)	728 (81.0)	**< 0.001**	144 (79.6)	162 (49.7)	**< 0.001**	97 (44.9)	209 (71.8)	**< 0.001**
7. Avoid taking public transportation	456 (89.9)	838 (93.2)	**< 0.021**	165 (91.2)	291 (89.3)	0.651	193 (89.4)	263 (90.4)	0.207
8. Avoid going to work	277 (54.6)	594 (66.1)	**< 0.001**	137 (75.7)	140 (42.9)	**< 0.001**	80 (37.0)	197 (67.7)	**< 0.001**
9. Avoid going to school or avoid letting children go to school	382 (75.3)	811 (90.2)	**0.001**	154 (85.1)	228 (69.9)	**0.024**	149 (69.0)	233 (80.1)	0.733
10. Avoid international travel	467 (92.1)	853 (94.9)	**0.032**	169 (93.4)	298 (91.4)	0.954	198 (91.7)	269 (92.4)	0.980
11. Avoid domestic or inter-city travel	440 (86.8)	805 (89.5)	0.076	165 (91.2)	275 (84.4)	0.091	174 (80.6)	266 (91.4)	**0.001**

Bold figures are significant

Only the most salient variables for social distancing have been reported in this table

^*^Pearson Chi-square test

### Satisfaction with government measures

Among participants who responded, HCWs were significantly more dissatisfied than NHCWs with the availability of Personal Protective Equipment (62% HCWs vs. 46% NHCWs, *p*-value < 0.001), testing kits (49% HCWs vs. 41% NHCWs, *p*-value 0.028), and screening facilities (54% HCWs vs. 42% NHCWs, *p*-value < 0.001). Please refer to Table 7

**Table 7:** Respondents’ satisfaction with government measures

Measures	HCWs	NHCWs	HCWs vs NHCWs	Healthcare students	Healthcare graduates	Students vs graduates	Frontline NHCWs	Backend NHCWs	Frontline vs backend
	*n* = 507	*n* = 589		*n* = 181	*n* = 326		*n* = 216	*n* = 290	
	No. (%)	No. (%)	*p*-value*	No. (%)	No. (%)	*p*-value*	No. (%)	No. (%)	*p*-value*
Screening facilities									
Satisfied/very satisfied	104 (20.6)	111 (18.8)	**< 0.001**	46 (25.6)	58 (17.9)	**0.016**	39 (18.2)	65 (22.4)	0.245
Neutral	117 (23.2)	198 (33.6)		44 (24.4)	73 (22.5)		49 (23.0)	68 (23.4)	
Unsatisfied/very unsatisfied	273 (54.2)	248 (42.2)		89 (49.4)	184 (56.8)		124 (57.9)	149 (51.4)	
Don’t know	10 (2.0)	32 (5.4)		1 (0.6)	9 (2.8)		2 (0.9)	8 (2.8)	
Laboratory services/testing kits									
Satisfied/very satisfied	109 (21.6)	124 (21.0)	**0.028**	50 (27.8)	59 (18.1)	**0.016**	41 (19.1)	68 (23.4)	0.344
Neutral	134 (26.5)	191 (32.4)		48 (26.7)	86 (26.5)		60 (27.9)	74 (25.6)	
Unsatisfied/very unsatisfied	248 (49.1)	243 (41.3)		78 (43.3)	170 (52.3)		111 (51.6)	137 (47.2)	
Don’t know	14 (2.8)	31 (5.3)		4 (2.2)	10 (3.1)		3 (1.4)	11 (3.8)	
Quarantine facilities									
Satisfied/very satisfied	134 (26.6)	157 (26.6)	**0.022**	56 (31.1)	78 (24.1)	**0.012**	56 (26.2)	78 (26.9)	0.602
Neutral	125 (24.8)	179 (30.4)		50 (27.8)	75 (23.1)		51 (23.8)	74 (25.5)	
Unsatisfied/very unsatisfied	228 (45.2)	223 (37.9)		69 (38.3)	159 (49.1)		103 (48.1)	125 (43.1)	
Don’t know	17 (3.4)	30 (5.1)		5 (2.8)	12 (3.7)		4 (1.9)	13 (4.5)	
Personal protective equipment									
Satisfied/very satisfied	86 (17.0)	100 (17.0)	**< 0.001**	39 (21.5)	47 (14.5)	0.072	29 (13.6)	57 (19.6)	0.076
Neutral	95 (18.8)	186 (31.6)		38 (21.0)	57 (17.6)		37 (17.3)	58 (19.9)	
Unsatisfied/very unsatisfied	311 (61.6)	268 (45.5)		102 (56.4)	209 (64.5)		144 (67.3)	167 (57.4)	
Don’t know	13 (2.6)	35 (5.9)		2 (1.1)	11 (3.4)		4 (1.8)	9 (3.1)	

Bold figures are significant

Only the most salient variables have been reported in this table. Percentages of categories “satisfied” and “very satisfied” were merged into category “satisfied/very satisfied”, and categories “unsatisfied” and “very unsatisfied” were merged into “unsatisfied/very unsatisfied”

^*^Mann–Whitney test

^†^Pearson Chi-square test

Similarly, compared to healthcare students, graduates were significantly more dissatisfied with screening facilities (57% graduates vs. 49% students, *p*-value 0.016), testing kits (52% graduates vs. 43% students, *p*-value 0.016), and quarantine facilities (49% graduates vs. 38% students, *p*-value 0.012).

## Discussion

This study shows that frontline HCWs, healthcare students, young people, women, and individuals with lower income in Pakistan were at a higher risk of psychological distress due to the pandemic. The findings indicate that almost three-quarters of both HCWs and NHCWs considered themselves susceptible to contract COVID-19. Healthcare graduates perceived themselves and their families to be more susceptible to COVID-19 and considered the disease to have a higher severity than students. However, students experienced depression symptoms more frequently.

Other studies in Pakistan conducted during the same time frame as our study corroborate our findings. One study in Karachi shows that HCWS experienced increased anxiety due to the fear of acquiring infection and transmitting it to their family members [18]. Other recent studies in Pakistan have mostly examined anxiety, depression, and stress, as well as perceptions about COVID-19 in other subsets of the population. For instance among university students in Pakistan the level of stress/anxiety was approximately 54% [19] which is very close to what we found in our study.

The latest Gallup survey (2021) in Pakistan shows that approximately half of the Pakistanis in urban areas continue to be worried if people around them do not wear masks in public [20]. This study is therefore unique in its targeting of Pakistan’s HCWs and NHCWs together in a comparative manner during the early stage of the pandemic. Our study further compares various subsets of population, viz. frontline versus backend health care workers and students versus graduates in health care. Moreover, a large survey assessing anxiety and depression symptoms in United States found that people were equally anxious at the start of the pandemic as they were in August 2021 [21]. Thus, while this study was conducted during the first wave of the pandemic, given the persistent levels of stress globally, the findings and recommendations are still valid.

A study from Hong Kong showed an even higher percentage of perceived susceptibility and severity towards COVID-19 [13]. This could be due to previous exposure to the SARS, and H1N1 outbreaks [22, 23]. Additionally, in this study frontline HCWs perceived themselves and their families to be more susceptible to COVID-19 than backend HCWs, while the latter perceived the disease to be more severe. Similarly, training status and clinical placement created differences in risk perception among the medical students of Iran [24]. Direct contact with COVID-19 patients is a major cause of concern among HCWs for themselves and their families. Greater perceived severity among backend workers on the other hand may be explained by the fact that since these workers are not seeing patients recover, their notion of disease severity is higher.

More than half of the respondents in the current study had some form of psychological distress (anxiety or depression). While our study reported that HCWs and NHCWs had similar anxiety levels, MEDS—frontline HCWs—in Italy reported higher anxiety levels in comparison to the general population [25]. This dissimilarity in anxiety levels observed between frontline HCWs and the general population in Italy and Pakistan can possibly be attributed to the high COVID-19 burden that Italian frontline HCWs (MEDS) were catering to at the time that study was conducted.

The results of this study show that the perceived impact of COVID-19 on daily routine was greater among frontline HCWs compared to the backend HCWs. Frontline HCWs (doctors and nurses) are involved in more direct patient care and have greater patient interaction. New protocols and added personal protective equipment (PPE) are focused on frontline workers which warrants a greater transition from the pre-pandemic life. Backend HCWs (pharmacists, dentists, physiotherapists, allied health sciences, and students) also had additions in their daily routine such as masks, social distancing, and hand sanitizing, however, these changes were less cumbersome. A study from China reported nurses experience more anxiety compared to doctors due to longer hours of direct patient care from the frontline [26].

This study highlights the greater burden of depressed mood for healthcare students than graduates. Existing data from Iran have shown students as a high-risk group for depression [27]. Experiences from the past epidemics also provide similar evidence [28, 29]. All educational institutes in Pakistan were closed during the duration of the study. Although educational institutes quickly adapted to online classes and virtual examinations, students took considerable time to adjust to new routines and methods of teaching. These interruptions in schedules, lack of physical interaction with peers, delayed graduation and social isolation may have contributed to the greater levels of depression.

Female gender, younger age, and the presence of COVID-19 related symptoms predicted increased psychological distress in HCWs while lower-income and presence of COVID-19 related symptoms predicted the same in NHCWs. Female gender has also been linked with greater anxiety levels in China and Iran [22, 23]. Factors contributing to distress during the COVID-19 pandemic might be the non-availability of personal protective equipment (PPE), uncertain employment conditions, lockdown, and work from home policies. COVID-19 has also increased the financial burden on households as many people struggle to run small businesses and maintain daily survival. The fear of not being able to fulfill the necessities may also be the reason why lower income levels are associated with increased anxiety levels.

Less than a third of HCWs and NHCWs were satisfied with the government’s measures to control COVID-19. HCWs, in comparison with NHCWs, were significantly more dissatisfied with the availability of PPEs and screening facilities. This is concerning, mainly because healthcare staff’s access to PPEs predicts lower distress levels, better physical health conditions, and more job satisfaction [30]. Therefore, the government must address these concerns, particularly among the HCWs who are the foot soldiers fighting the pandemic.

This is a novel study accounting for differences in experiences among health care and non-health care workers and other subgroups. However, there were a few limitations to this study. An all-encompassing definition of HCWs was used in order to evaluate risk perceptions and psychological distress due to COVID-19 in the maximum number of people involved with the healthcare setup. Similarly, we used a broad criteria to define frontline HCWs, taking into account possible categories of HCWs that could be involved in direct patient care. However, despite doing a comparative analysis of frontline and backend HCWs to better describe results among HCWs, our findings may suggest a greater level of anxiety in frontline HCWs compared to other studies that used other definitions of HCWs. Among frontline health care workers, we had to exclude junior ancillary staff who though are involved in direct patient handling have limited literacy and internet access to respond to such online surveys. Moreover, in this study, most of the respondents were aged less than 35 years, which may not accurately represent the older population who are at greater risk for contracting COVID-19. Nevertheless, as the majority of the population in Pakistan is below the age of 30 years, the non-health care respondents are likely to represent the perceptions of the literate general population in Pakistan. Additionally, while analysis has been done about gender and prediction of distress, it cannot be commented if certain genders were more likely to be in certain roles which could have possibly skewed the findings. Further, this study was a cross-sectional study, and future cohort studies are recommended to assess relative risks and predictive value of perceived severity and susceptibility, adoption of precautions, and respondents' satisfaction with government measures as independent variables impacting psychological outcomes. Indeed early evidence from China suggests that the odds of psychological distress are low among those who adopt precautionary measures [31].

To decrease the level of psychological distress, hospital administrators should implement policies to target the mental well-being of the HCWs, such as the suggested development and implementation of an urgent psychological crisis intervention model (PCIM) through the medium of internet technology [32, 33]. This involves integrating teams of physicians, psychiatrists, and social workers to deliver early psychological intervention to patients, families, and medical staff. Hospital staff dealing with COVID-19 patients should also be monitored regularly to avoid burnout. One study conducted among HCWs in Karachi recommended that developing a safe hospital environment, adequate training, and supportive management can ameliorate stress among health workers [18]. HCWs’ positive perception of personal protection is important when managing patients with COVID-19. It is therefore understandable that a study from Pakistan advocates reducing anxiety, workload and family strain among HCWs treating COVID-19 patients at the frontline [34].

Another study found that HCWs who perceive organizational support experience less job-related stress compared to those who did not perceive such support [35]. Incentives such as financial bonuses and paid leave should be provided. The government should ensure the provision of PPE, testing kits, and screening facilities to increase the satisfaction levels of HCWs in particular and the public at large. Furthermore, implementing these strategies may also contribute to mitigating the spread of COVID-19. The better the disease is controlled, the lesser will be the psychological morbidity and adverse impact it has on people’s mental health.

## Conclusion

HCWs and NHCWs both have high levels of perceived susceptibility and severity along with increased psychological distress. This study identified vulnerable groups such as frontline HCWs, healthcare students, younger aged people, women, and individuals with lower income to be at a higher risk of psychological distress. Further studies need to investigate a direct link between HCWs and the development of COVID-19 infection to quantify the infection risk. This study adds to a growing body of literature suggesting a rising burden of anxiety and depression among health care workers and the need to promote their mental well-being.

## Supplementary Information

**Additional file 1.** Questionnaire.

**Additional file 2.** Data.

## Data Availability

The datasets used and/or analyzed during the current study are available from the corresponding author on reasonable request.

## Abbreviations

AKU: Aga Khan University
AKUH: Aga Khan University Hospital
COVID-19: Coronavirus disease of 2019
HADS: Hospital Anxiety and Depression Scale
HCWs: Healthcare workers
NHCWs: Non-healthcare workers
PCIM: Psychological crisis intervention model
PPE: Personal protective equipment
SARS: Severe acute respiratory syndrome
SARS-CoV: SARS-coronavirus
SPSS: Statistical Package for the Social Sciences

## References

[1] Lu H, Stratton CW, Tang Y-W (2020). Outbreak of pneumonia of unknown etiology in Wuhan, China: the mystery and the miracle. J Med Virol.

[2] Tran K, Cimon K, Severn M, Pessoa-Silva CL, Conly J (2012). Aerosol generating procedures and risk of transmission of acute respiratory infections to healthcare workers: a systematic review. PLoS ONE.

[3] Weber DJ, Rutala WA, Schaffner W (2010). Lessons learned: protection of healthcare workers from infectious disease risks. Crit Care Med.

[4] Hui DSC, Chan PKS (2010). Severe acute respiratory syndrome and coronavirus. Infect Dis Clin N Am.

[5] Balicer RD, Omer SB, Barnett DJ, Everly GS (2006). Local public health workers’ perceptions toward responding to an influenza pandemic. BMC Public Health.

[6] Tian C, Lovrics O, Vaisman A, Chin KJ, Tomlinson G, Lee Y (2021). Risk factors and protective measures for healthcare worker infection during highly infectious viral respiratory epidemics: a systematic review and meta-analysis. Infect Control Hosp Epidemiol.

[7] Lai J, Ma S, Wang Y, Cai Z, Hu J, Wei N (2020). Factors associated with mental health outcomes among health care workers exposed to coronavirus disease 2019. JAMA Netw Open.

[8] Aoyagi Y, Beck CR, Dingwall R, Nguyen-Van-Tam JS (2015). Healthcare workers’ willingness to work during an influenza pandemic: a systematic review and meta-analysis. Influenza Other Respir Viruses.

[9] Huang Y, Zhao N (2020). Generalized anxiety disorder, depressive symptoms and sleep quality during COVID-19 outbreak in China: a web-based cross-sectional survey. Psychiatry Res.

[10] Rajkumar RP (2020). COVID-19 and mental health: a review of the existing literature. Asian J Psychiatry..

[11] Mohindra R, Ravaki R, Suri V, Bhalla A, Singh SM (2020). Issues relevant to mental health promotion in frontline health care providers managing quarantined/isolated COVID19 patients. Asian J Psychiatr.

[12] Huang JZ, Han MF, Luo TD, Ren AK, Zhou XP (2020). Mental health survey of 230 medical staff in a tertiary infectious disease hospital for COVID-19. Chin J Ind Hyg Occup Dis.

[13] Kwok KO, Li KK, Chan HH, Yi YY, Tang A, Wei WI. Community responses during the early phase of the COVID-19 epidemic in Hong Kong: risk perception, information exposure and preventive measures. Emerg Infect Dis. 2020. https://wwwnc.cdc.gov/eid/article/26/7/20-0500_article. 10.3201/eid2607.200500 PMC7323558 32298227

[14] Djukanovic I, Carlsson J, Årestedt K (2017). Is the Hospital Anxiety and Depression Scale (HADS) a valid measure in a general population 65–80 years old? A psychometric evaluation study. Health Qual Life Outcomes.

[15] Hasan SM, Rehman A, Zhang W (2021). Who can work and study from home in Pakistan: evidence from a 2018–19 nationwide household survey. World Dev.

[16] Social P. Living standards measurement survey (PSLM). Statistics Division, Government of Pakistan. 2004;5.

[17] Hulley SB (2007). Designing clinical research.

[18] Feroz AS, Pradhan NA, Ahmed ZH, Shah MM, Asad N, Saleem S (2021). Perceptions and experiences of healthcare providers during COVID-19 pandemic in Karachi, Pakistan: an exploratory qualitative study. BMJ Open.

[19] Raja HZ, Saleem MN, Saleem T, Rashid H, Ehsan S, Hakeem S, et al. Perceived stress levels in Pakistani dental students during COVID-19 lockdown. Eur J Dent Oral Health. 2020;1(4). https://ejdent.org/index.php/ejdent/article/view/14. Accessed 1 Oct 2021.

[20] Gallup Pakistan. Coronavirus attitude tracker survey Pakistan: wave 10 results. 2021;64.

[21] National Center for Health Statistics, CDC. Mental Health—Household Pulse Survey—COVID-19. 2021. https://www.cdc.gov/nchs/covid19/pulse/mental-health.htm. Accessed 21 Sept 2021.

[22] Coughlin SS (2012). Anxiety and depression: linkages with viral diseases. Public Health Rev.

[23] Chang C (2012). News coverage of health-related issues and its impacts on perceptions: Taiwan as an example. Health Commun.

[24] Taghrir MH, Borazjani R, Shiraly R (2020). COVID-19 and Iranian medical students; a survey on their related-knowledge, preventive behaviors and risk perception. Arch Iran Med.

[25] Simione L, Gnagnarella C. Differences between health workers and general population in risk perception, behaviors, and psychological distress related to COVID-19 spread in Italy. PsyArXiv. 2020. https://psyarxiv.com/84d2c. 10.3389/fpsyg.2020.02166 PMC7499804 33013555

[26] Zhu J, Sun L, Zhang L, Wang H, Fan A, Yang B (2020). Prevalence and influencing factors of anxiety and depression symptoms in the first-line medical staff fighting against COVID-19 in Gansu. Front Psychiatry.

[27] Jahanshahi AA, Dinani MM, Madavani AN, Li J, Zhang SX (2020). The distress of Iranian adults during the Covid-19 pandemic—more distressed than the Chinese and with different predictors. Brain Behav Immun.

[28] Wong TW, Gao Y, Tam WWS (2007). Anxiety among university students during the SARS epidemic in Hong Kong. Stress Health.

[29] Main A, Zhou Q, Ma Y, Luecken LJ, Liu X (2011). Relations of SARS-related stressors and coping to Chinese college students’ psychological adjustment during the 2003 Beijing SARS epidemic. J Couns Psychol.

[30] Zhang SX, Liu J, Afshar Jahanshahi A, Nawaser K, Yousefi A, Li J (2020). At the height of the storm: healthcare staff’s health conditions and job satisfaction and their associated predictors during the epidemic peak of COVID-19. Brain Behav Immun.

[31] Wang C, Pan R, Wan X, Tan Y, Xu L, Ho CS (2020). Immediate psychological responses and associated factors during the initial stage of the 2019 coronavirus disease (COVID-19) epidemic among the general population in China. Int J Environ Res Public Health.

[32] Rana W, Mukhtar S, Mukhtar S (2020). Mental health of medical workers in Pakistan during the pandemic COVID-19 outbreak. Asian J Psychiatr.

[33] Sandesh R, Shahid W, Dev K, Mandhan N, Shankar P, Shaikh A. Impact of COVID-19 on the mental health of healthcare professionals in Pakistan. Cureus J Med Sci. 2020. https://www.cureus.com/articles/35257-impact-of-covid-19-on-the-mental-health-of-healthcare-professionals-in-pakistan. 10.7759/cureus.8974 PMC7402435 32775056

[34] Mahmood QK, Jafree SR, Jalil A, Nadir SMH, Fischer F (2021). Anxiety amongst physicians during COVID-19: cross-sectional study in Pakistan. BMC Public Health.

[35] Zandi G, Shahzad I, Farrukh M, Kot S (2020). Supporting role of society and firms to COVID-19 management among medical practitioners. Int J Environ Res Public Health.

