# Supplementary material for: Perceived risk and distress related to COVID-19 in healthcare versus non-healthcare workers of Pakistan: a cross-sectional study
Source: Hum Resour Health. 2022 Jan 22;20:11. doi: 10.1186/s12960-022-00705-4 (PMC8783178; doi:10.1186/s12960-022-00705-4)
Supplement: Supplementary file 1 — Additional file 1. Questionnaire. [file 12960_2022_705_MOESM1_ESM.pdf]

“Risk perceptions and anxiety level amongst public and health professionals: what can the health system offer during COVID -19 in Pakistan?”

Introduction and Informed Consent

You are being asked to participate in this survey conducted by The Aga Khan University, which aims to study the risk perceptions and anxiety levels towards Coronavirus Disease 2019 (COVID-19) amongst general public and health professionals.

We will be asking a series of simple questions and do not anticipate any risk or discomfort to you. It will take about 20 minutes to complete this survey and your participation is voluntary. Information collected from this survey will be kept confidential. You will not be provided any financial incentive to take part in the research.

Your responses will help to develop local/national policies and to tackle the COVID-19 pandemic in Pakistan. This study has been approved by Aga Khan University Ethical Review Committee. For any queries, please contact the research team at (insert new Gmail ID here).

Only individuals aged 18 or above who are permanently living in Pakistan can participate in this survey.

Are you willing to participate in this study?

a. • Yes →Next question
b. • No → End

Are you 18 years old or above?

a. • Yes →Next question
b. • No → End

In the last month, on an average, did you live in Pakistan for at least 5 days per week?

a. • Yes →Next question
b. • No → End

Are you a healthcare professional with a formal basic qualification (Bachelors/Diploma), or a current student, in *Medicine*, *Nursing*, *Pharmacy*, *Dentistry*, *Physiotherapy*, *Laboratory Technology*, *Allied Health Sciences* or *Homeopathy*?

a. • Yes → Include “Additional Questionnaire for HCWs”
b. • No→ Do not include “Additional Questionnaire for HCWs”

Section A. General Information

1. What is your gender?
   a. • Male
   b. • Female
   c. • Prefer not to disclose

2. Which age group, do you belong to?
   a. • 18-24years
   b. • 25-34 years
   c. • 35-44 years
   d. • 45-54years
   e. • 55-64 years
   f. • 65-74 years
   g. • 75 years or above
   h. • Prefer not to disclose

3. What is your highest level of education?
   a. • No schooling/Pre-primary
   b. • Primary (Grade 1-5)
   c. • Secondary (Grade 6-8)
   d. • Matric/O-Levels
   e. • Intermediate/A-Levels/ International Baccalaureate
   f. • Post-intermediate: Diploma/Certificate
   g. • Bachelor
   h. • Master
   i. • PhD
   j. • Other, (please specify: ___________)
   k. • Prefer not to disclose

4. With reference to the past month, what is your household income?
   a. • PKR 20,000 or below
   b. • PKR 20,001 – PKR 40,000
   c. • PKR 40,001 – PKR 60,000
   d. • PKR 60,001 – PKR 80,000
   e. • PKR 80,001 – PKR 100,000
   f. • PKR 100,001 – PKR 120,000
   g. • ≥ PKR 120,001
   h. • Prefer not to disclose

5. Which city of Pakistan do you permanently reside in?

- a. • Islamabad
- b. • Karachi (If yes proceed to 5a)
- c. • Lahore
- d. • Peshawar
- e. • Quetta
- f. • Other, please specify (__________________)

5a. Which district of Karachi do you reside in?

- a. • District Central
- b. • District East
- c. • District Korangi
- d. • District Malir
- e. • District South
- f. • District West

6. In the last month, did you travel outside your city of permanent residence in Pakistan?

- a. • Yes → (if yes proceed to 6a)
- b. • No

6a. Where did you travel to? (Multiple options allowed)

- a. • Sindh
- b. • Baluchistan
- c. • Punjab
- d. • Khyber Pakhtunkhwa
- e. • Azad Kashmir
- f. • Gilgit Baltistan
- g. • Outside Pakistan (If yes proceed to 6b)
- h. • Prefer not to disclose

6b. Which foreign countries did you travel to in the last month?

- a. • Asia
- b. • China
- c. • Europe (Other than UK, Italy, Spain)
- d. • Iran
- e. • Italy
- f. • Saudi Arabia
- g. • Spain
- h. • United Kingdom
- i. • United States of America
- j. • Others, please specify _________________

Section B. Health Status

7. How do you perceive your health status?
   a. • Very good
   b. • Good
   c. • Fair
   d. • Bad
   e. • Very bad
   f. • Prefer not to disclose

8. In the past 14 days, did you visit a medical practitioner?
   a. • No
   b. • Yes, (if yes proceed to 8a)

   8a. What kind of practitioner did you visit?
      a. • Private practitioner/clinic/
      b. • Private hospital
      c. • Government hospital
      d. • Homeopathic practitioner
      e. • Other, (please specify:________)
      f. • Prefer not to say
      g. • Outside Pakistan (If yes proceed to 6b)
      h. • Prefer not to disclose

9. In the past 14 days, did you experience any symptom(s) of illness?
   (for example: fever, sore throat, cough etc.)
   a. • Yes → Q10
   b. • No→ Q11

10. What symptoms did you have? (Multiple answers allowed)
   a. • Cough
   b. • Difficulty in breathing
   c. • Dizziness
   d. • Headache
   e. • Muscle pain/Body ache
   f. • Persistent fever (body temperature ≥ 38°C for at least one day)
   g. • Runny nose
   h. • Shivering
   i. • Sore throat
   j. • Others, (please specify: ________)

Section C. Perceived Susceptibility and Severity

11. If no preventive measure is taken, how likely do you think you will be infected with COVID-19?

a. • Very likely
b. • Likely
c. • Neutral
d. • Unlikely
e. • Very unlikely
f. • Prefer not to disclose

12. If no preventive measure is taken, how likely do you think your family member(s) will be infected with COVID-19?

a. • Very likely
b. • Likely
c. • Neutral
d. • Unlikely
e. • Very unlikely
f. • Prefer not to disclose

13. How severe do you think are the symptoms caused by COVID-19?

a. • Very severe
b. • Severe
c. • Neutral
d. • Not severe
e. • Not severe at all
f. • Prefer not to disclose

14. In your opinion, how high, is the chance of survival for COVID-19 patients?

a. • Very high
b. • High
c. • Neutral
d. • Not high
e. • Not high at all
f. • Prefer not to disclose

Section D. Knowledge of COVID-19

15. How well do you know about COVID-19?
   a. • Not well at all
   b. • Not well
   c. • Normal
   d. • Well
   e. • Very well
   f. • Prefer not to disclose

16. How likely is COVID-19 transmitted through the following routes?

		1.Very likely	2.Likely	3.Neutral	4.Unlikely	5.Very unlikely
A	Face-to-face conversation with a COVID-19 patient who **has no symptoms** (without direct physical contact)					
b	Face-to-face conversation with a COVID-19 patient who **has symptoms** (without direct physical contact)					
c	Direct physical contact with a COVID-19 patient who **has no symptoms**					
d	Having body touch with a COVID-19 patient **who has symptoms**					
e	Respiratory droplets					
f	Airborne droplets (when infected persons cough or sneeze)					
g	Contaminated environment					
h	Visiting meat shops/markets					
i	Consumption of seafood/ meat					
j	Consumption/use of products imported from China					

17. To combat COVID-19, do you adopt the following precautions?

		Yes	No	Not Applicable
a	Wear face masks			
b	Wash hands frequently (With soap or hand sanitizer)			
c	Disinfecting floors and tables at home (with phenyl products)			
d	Cover nose and mouth when sneezing or coughing			
e	Avoid contacting people who have fever or respiratory symptoms			
f	Avoid contacting people who have been traveling abroad within one month			
g	Avoid going out			
h	Avoid crowded areas			
i	Avoid going to meat shops/market			
j	Avoid going to hospital or clinic			
k	Avoid taking public transportation			
l	Avoid going to work			
m	Avoid going to school or avoid letting kids go to school			
n	Avoid social events			
o	Avoid international travel			
p	Avoid domestic or inter-city travel			

Section E. Information Sources

18. Are you constantly alert about the disease progression of COVID-19?
   a. • Yes
   b. • No

19. Through which channels do you know about COVID-19? (Multiple answers allowed)
   a. • Newspaper
   b. • Magazine
   c. • Radio
   d. • Television
   e. • Internet/Websites
   f. • Social media platforms (e.g. Facebook, Instagram, Twitter, WhatsApp)
   g. • Doctor
   h. • Family or friends
   i. • Others (please specify: ______________________)

20. What types of information about COVID-19 do you want to receive? (Multiple answers allowed)
   a. • Symptoms / How to know if I am infected with COVID-19
   b. • Current situation: number of infected cases
   c. • Current situation: distribution of cases
   d. • What to do if infected with COVID-19
   e. • Preventive measures
   f. • The intervention measures against COVID-19 enacted by the Provincial or Federal Government
   g. • Others, (please specify: _______________)

21. How reliable, do you think, are these information sources?

		1.Very reliable	2.Reliable	3.Neutral	4.Unreliable	5.Very unreliable
a	Newspaper					
b	Magazine					
c	Radio					
d	Television					
e	Official websites, like the Government					
f	Unofficial websites					
g	Social media platforms (WhatsApp, Facebook, Instagram)					
h	Your doctor					
i	Your family or friends					

Section F. Psychosocial

For Q22-34, please select the answer which best describes your feeling in the *past* week. Do not take too long over your replies; your immediate response is the best.

22. I feel tense
    a. • Most of the time
    b. • A lot of the time
    c. • From time to time, occasionally
    d. • Not at all

23. I get a sort of frightened feeling as if something awful is about to happen.
    a. • Very definitely and quite badly
    b. • Yes, but not too badly
    c. • A little, but it doesn't worry me
    d. • Not at all

24. Worrying thoughts go through my mind.
    a. • A great deal of the time
    b. • A lot of the time
    c. • From time to time, but not too often
    d. • Only occasionally

25. I can sit at ease and feel relaxed.
    a. • Definitely
    b. • Usually
    c. • Not Often
    d. • Not at all

26. I get a sort of frightened feeling
    a. • Not at all
    b. • Occasionally
    c. • Quite often
    d. • Very often

32. I feel restless and cannot sit still
    a. • Very much indeed
    b. • Quite a lot
    c. • Not very much
    d. • Not at all

27. I get sudden feelings of panic.
    a. • Very often indeed
    b. • Quite often
    c. • Not very often
    d. • Not at all

28. I still enjoy the things I used to enjoy.
   a. • Definitely as much
   b. • Not quite as much
   c. • Only a little
   d. • Hardly at all

29. I can laugh and see the funny side of things.
   a. • Very often indeed
   b. • Quite often
   c. • Not very often
   d. • Not at all

30. I feel cheerful.
   a. • Not at all
   b. • Not often
   c. • Sometimes
   d. • Most of the time

31. I feel as if I am slowed down.
   a. • Nearly all the time
   b. • Very often
   c. • Sometimes
   d. • Not at all

32. I have lost interest in my appearance.
   a. • Definitely
   b. • I don't take as much care as I should
   c. • I may not take quite as much care
   d. • I take just as much care as ever

33. I look forward to things with enjoyment
   a. • As much as I ever did
   b. • Rather less than I used to
   c. • Definitely less than I used to
   d. • Hardly at all

34. I can enjoy a good book or radio or TV programme.
   a. • Often
   b. • Sometimes
   c. • Not often
   d. • Very often

35. Does COVID-19 affect your daily life?
   a. • Not at all
   b. • A bit
   c. • Greatly
   d. • Not sure
   e. • Prefer not to disclose

36. Are you worried about COVID-19?
   a. • Yes→Q37
   b. • No→Q38

37. Regarding COVID-19, how worried are you?
   a. • Worried a lot
   b. • Worried
   c. • Neutral
   d. • Not worried
   e. • Not worried at all
   f. • Prefer not to disclose

38. **The following questions concern social distancing in the last *two weeks* and your reaction to the phase. Please rate the extent to which agree with each statement:**

	**Strongly Disagree**	**Disagree**	**Neutral**	**Agree**	**Strongly Agree**	**Prefer not to disclose**
My family might contract the disease						
COVID-19 will affect my job						
COVID-19 will affect my personal life						
I might contract COVID-19 if one of my family members test positive for the disease						
COVID-19 has affected my sleeping pattern						
COVID-19 has affected my eating habits						

Section G. Health Delivery Services

44. Are you aware of the healthcare services for COVID-19 that are being provided by your Provincial Government?

a. • Yes
b. • No

45. What types of healthcare services for COVID-19 are currently being provided by your Provincial Government? (Multiple options allowed)

a. • Isolation ward
b. • Laboratory testing
c. • PPE among healthcare workers
d. • Quarantine facilities
e. • Screening
f. • Treatment facilities
g. • Ventilators
h. • Others (please specify: _________________)

46. Do you know where the healthcare services for COVID-19 provided by the Government are available locally?

a. • Yes
b. • No

47. What are the other measures being taken by the Provincial and Federal Government to control COVID-19?

a. • Closure of airlines for domestic travel
b. • Closure of airlines for international travel
c. • Closure of government offices
d. • Closure of private and public transport between cities
e. • Closure of private offices
f. • Closure of public transport facilities within your city
g. • Closure of schools
h. • Limiting the private travel within your city
i. • Others, (please specify_____)

Additional Questionnaire for HCWs

48. How satisfied are you with the individual measures taken by the Government to control COVID- 19? (Multiple options allowed)

Measure	Very Unsatisfied	Unsatisfied	Neutral	Satisfied	Very Satisfied
**Awareness campaign**					
**Economic Relief Package**					
**Lockdown/ Social distancing strategies**					
**Screening facilities**					
**Lab Services/ Testing Kits**					
**Isolation Wards/ Quarantine facilities**					
**PPEs**					
**Ventilators**					

49. Would you like to recommend any policy/policies to the Government to control COVID-19 in your province? (Please mention your suggestions below)

Thank you for completing the survey!

Please feel free to share this survey link with your family, friends, and colleagues through Facebook, Twitter, LinkedIn, WhatsApp and/or any social media platforms.

Kindly enter your email address and name in the fields below if you would like to receive the following benefits associated with the completion of this survey:

- *Receive an e-certificate of participation*
- *Stay informed about future developments and interventions related to COVID-19*
- *Obtain a referral to relevant channels in the healthcare system (only applicable for participants that are found to be anxious, if they so opt for)*

Email: ___________________________

Name: ___________________________

************************ The End **************************
